# Organizing and Improving Outcomes for Changing Teaching in Neurological Surgery Residency Using Innovation

**DOI:** 10.7759/cureus.108447

**Published:** 2026-05-07

**Authors:** Melissa Miulli, Christopher Wong, Jose Gonzalez, Sanjay C Rao, Vartan Tashjian, Alice Wang, Paras Savla, Dan E Miulli

**Affiliations:** 1 Education, California Baptist University, Riverside, USA; 2 Neurological Surgery, Riverside University Health System Medical Center, Moreno Valley, USA

**Keywords:** innovation, neurosurgical residency, osteopathic, point system, promotion, remediation

## Abstract

Background: Neurosurgical training is uniquely constrained by prolonged duration, limited case volumes, and high-stakes decision-making that demands simultaneous mastery of technical skills, clinical judgment, professionalism, and communication. Traditional assessment systems often detect late deficits and provide insufficiently granular feedback for early, individualized remediation.

Methods: We implemented an integrated educational performance framework in an Accreditation Council for Graduate Medical Education (ACGME) osteopathic-recognized neurosurgery residency consisting of: (1) a point-based quarterly promotion system capturing required clinical, scholarly, and professional activities; (2) a resident-generated multiple-choice question and quiz program linked to didactic content and in-service exam domains; (3) simulation-enhanced technical training using cadavers, three-dimensional (3D)-printed models, and virtual reality; and (4) a predictive analytics dashboard that synthesizes longitudinal performance signals to identify strengths and emerging risks early.

Results: Across trainees, quarterly point totals and quiz performance provided actionable early markers of engagement and knowledge acquisition. Residents falling ≥10% below quarterly point thresholds on two consecutive quarters were reliably identified for goal-oriented success plans, enabling targeted remediation or, when necessary, determination of inability to meet program requirements. Quiz participation and scores are tracked with bi-monthly examinations and annual in-service performance, allowing topic-specific coaching and peer-mentoring by high performers. Simulation interventions demonstrated measurable skill acquisition and procedural efficiency improvements consistent with prior published program outcomes.

Conclusions: A novel combined point-quiz-simulation framework, strengthened by predictive analytics, creates a closed-loop competency system that detects deficits early, amplifies strengths, and aligns resident and faculty actions to program mission, milestones, and patient-centered outcomes. This approach is feasible, transferable, and may help address regional neurosurgical access gaps by accelerating the development of safe, independent surgeons while preserving wellness and professionalism. A core finding of this manuscript is that measurable structure improves resident function.

## Introduction

Neurosurgery is among the most specialized disciplines in medicine, with a comparatively small workforce and a training pathway that is longer and more resource-intensive than any other specialty. In the United States, estimates suggest there are roughly 3,500-3,800 actively practicing, board-certified neurosurgeons, creating a persistent mismatch between community need and available expertise [1]. Traditional workforce planning models have suggested that one neurosurgeon is required for approximately 90,000-100,000 people, and some analyses propose that an even more favorable ratio (near 1:80,000) may be needed as the population ages and obesity-related degenerative disease increases. In contrast, the primary-care workforce is far larger, and optimal ratios may be as low as 1 physician per 5,000 patients. This disparity underscores why access gaps in neurosurgical care are felt acutely in many regions, including Southern California, where rapid population growth, rising trauma volumes, and complex chronic disease amplify demand.

The neurosurgical workforce pipeline is constrained by the length and intensity of training. After undergraduate and medical school education, residents complete seven years of residency training, with many pursuing fellowship training thereafter. This duration is a major financial and personal commitment, especially given high educational debt burdens that commonly approach or exceed $250,000 [2]. The extended pathway can deter applicants and contribute to workforce shortages, particularly in underserved or geographically dispersed regions. Because neurosurgeons are relatively few and their clinical obligations are high, the "teaching time" required to educate residents competes directly with operative and consultative demands.

Neurosurgery also presents unique educational challenges related to case volume and complexity. Smaller community hospitals, rural settings, and many suburban regions have limited neurosurgical presence and case diversity, reducing opportunities for early exposure [3]. Even within high-volume centers, certain procedures occur infrequently, making it difficult for residents to achieve sufficient repetition to develop true expertise. For these reasons, neurosurgical education has historically relied on an apprenticeship model, in which techniques and judgment are transmitted through direct observation, supervised performance, and accumulated clinical experience [4].

However, the apprenticeship model, while indispensable, has important limitations. Neurosurgery contains fewer Level I data than many other disciplines, and best practices may be shaped by expert consensus and iterative refinement rather than definitive trials [5]. In this setting, residency programs must ensure that clinical tradition is complemented by rigorous educational design, objective assessment, and mechanisms that reliably identify learner strengths and weaknesses early enough to redirect growth. Modern competency-based medical education frameworks emphasize this need: trainees must be assessed longitudinally across multiple domains, coached toward milestones, and supported with structured remediation when gaps emerge [6].

Within this larger framework, Riverside University Health System (RUHS) has developed a structured resident promotion point system that operationalizes accountability across the full spectrum of required activities. The system makes expectations explicit, measures completion and performance with transparent thresholds, and uses quarterly feedback to identify residents who are excelling and those who are drifting off course. In doing so, it preserves the strengths of apprenticeship - personal mentorship, real-time coaching, and professional identity formation - while adding a reproducible, technology-enabled method to detect and address gaps early [7]. This blend of tradition, competency-based assessment, and innovation provides the foundation for the program’s broader strategy to organize and improve educational outcomes.

The RUHS program also applies established quality improvement methodologies such as Plan-Do-Study-Act cycles, Lean process improvement, and Six Sigma principles to the educational environment itself [8]. Translating these methods to graduate medical education creates a disciplined way to define goals, measure performance, test interventions, and sustain improvement. Within neurosurgery, where the stakes are high and the learning curve is steep, an intentional, data-driven approach is essential to support both excellence and accountability.

Technology is central to this approach. Residency management platforms allow the program to integrate duty hour tracking, case logs, operative assessments, and milestone mapping into a longitudinal record that can be reviewed by residents, faculty, and the Clinical Competency Committee (CCC) [9]. Digital dashboards enable the program to recognize patterns that might be missed in episodic evaluations, such as emerging deficiencies in procedural exposure or deterioration in wellness indicators. When combined with structured feedback conversations, these tools create a continuous quality improvement loop for both learner development and program improvement.

The RUHS Neurosurgery Residency addresses these challenges by integrating mission-aligned competencies, structured curricula, and technology-enabled assessment. Importantly, assessment is designed to be actionable: data are used not merely to document competence but also to identify early divergence from expected trajectories as well as to trigger targeted coaching, additional operative exposure, focused simulation, or individualized study plans [10].

The objectives of this study are fourfold: (1) to describe the structure and implementation of a point-based quarterly promotion system; (2) to evaluate the predictive validity of a resident-generated quiz and question-writing program; (3) to characterize simulation-enhanced technical training outcomes; and (4) to present a predictive analytics framework for early identification of resident strengths and emerging deficits.

## Materials and methods

Structured point system

Promotion within neurosurgical residency requires more than simply meeting minimum clinical responsibilities. The RUHS Neurosurgery Residency employs a structured point-based system (Table 1) that not only tracks completion of required activities but also highlights individual strengths and weaknesses, guiding both residents and faculty in tailoring development plans that promote long-term success.

**Table 1:** Point system. ABNS: American Board of Neurological Surgery; AOA: American Osteopathic Association; RUHS: Riverside University Health System.

Activity	Points
Attending lectures on time. There are 50 lecture sessions; residents are required to attend 80% (40) - 4 pts each lecture on time, with a minus one for each 15 min late. Residents can receive 1 point if they watch the lecture video within 14 days. If residents do not receive 160 points, then they will lose 40 pts.	160; bonus 40
Lecture preparation: turn in lecture to faculty Monday, three weeks ahead of time; bonus 1 if finished and added to the neurosurgery internet database Monday, one week before lecture. If not sent to faculty Monday two weeks ahead of date: −2 points; if not sent to faculty and placed on neurosurgery internet database by Monday, one week ahead of lecture: another −3 points.	1 bonus
Evaluation rotation completion (1 point per quarter completed on time. If residents do not complete the evaluation by the following quarter, the due date residents will lose 20 pts. If residents do not complete by July 10, residents will lose 40 pts).	4
Evaluation attending completion (1 point per quarter completed on time. If residents do not complete evaluation by following quarter, due date resident will lose 20 pts. If residents do not complete by July 10, resident will lose 40 pts).	4
Evaluation resident by faculty completion (1 point per quarter completed on time. If residents do not complete evaluation by following quarter, due date resident will lose 20 pts. If residents do not complete by July 10, resident will lose 40 pts).	4
Evaluation handoff completion (1 point per quarter completed on time. If residents do not complete evaluation by following quarter, due date resident will lose 20 pts. If residents do not complete by July 10, resident will lose 40 pts).	4
Evaluation history and physical completion (1 point per quarter completed on time. If residents do not complete evaluation by following quarter, due date resident will lose 20 pts. If residents do not complete by July 10, resident will lose 40 pts).	4
Evaluation osteopathic manipulative treatment completion (1 point per quarter completed on time. If residents do not complete evaluation by following quarter, due date resident will lose 20 pts. If residents do not complete by July 10, resident will lose 40 pts).	4
Evaluation operative/procedure completion (1 point per quarter completed on time. If residents do not complete evaluation by following quarter, due date resident will lose 20 pts. If residents do not complete by July 10, resident will lose 40 pts).	4
Evaluation grand rounds presentation completion (1 point per grand rounds presentation and evaluation completed on time. If residents do not complete evaluation by following quarter, due date resident will lose 20 pts. If residents do not complete by July 10, resident will lose 40 pts).	4
Evaluation peer completion (2 points per biannual completed on time. If residents do not complete evaluation by following biannual, due date resident will lose 40 pts).	4
Evaluation 360 completion (2 points per biannual completed on time. If residents do not complete evaluation by following biannual, due date resident will lose 40 pts).	4
Evaluation patient completion (2 points per biannual completed on time. If residents do not complete evaluation by following biannual, due date resident will lose 40 pts).	4
Evaluation self-completion (2 points per biannual completed on time. If residents do not complete evaluation by following biannual, due date resident will lose 40 pts).	4
Annual biannual evaluation completion (2 points per biannual completed on time. If residents do not complete evaluation by following biannual, due date resident will lose 40 pts).	4
AMA GCEP completion (4 points per first RUHS rotation - course completed on time or March 31 if no RUHS. If residents do not complete course on time, residents will lose 40 points).	4
IHI completion (4 points per first RUHS rotation - course completed on time or March 31 if no RUHS. If residents do not complete course on time, residents will lose 40 points).	4
Book club completion (4 points per first RUHS rotation - course completed on time. If residents do not complete courses on time, residents will lose 40 points unless no RUHS).	4
Continuity clinic 48 half days (if residents do not complete 48 half days, resident will lose 40 points).	4
Mentor meeting (1 point per quarter if all three monthly meetings are completed. If residents do not obtain 3 pts in category, resident will lose 40 pts for the year).	4
In-service exam performance at or above level (PGY6/7: 70; PGY5: 65; PGY4: 60; PGY3: 55; PGY2: 50; PGY1: 45). If not obtained score for resident year, then lose 16 pts. If not obtained a score of 70 by graduation, resident will lose 60 pts that year and cannot graduate.	16
In-service exceptional score above 80% or ABNS above 500 receives bonus points and two extra personal days off.	Bonus 30
Weekly quiz 3:00-3:15, 1 point completing on time, 1 point for 100%.	1 and bonus 1
Cadaver class (2 points for each class participated arriving within 15 min of assigned time. If residents do not obtain 4 pts in category, resident will lose 40 pts for the year.)	4; bonus 2
Meeting scholarly activity timelines (2 points per quarter timeline met. This includes poster presentation. If residents do not present poster, resident will lose 2 points in that quarter; 2 for third at RUHS; 2 for fourth at other hospital. If residents do not obtain 8 pts in category, resident will lose 40 pts for the year).	8
Publishing PubMed ID (if residents do not publish with a PubMed ID, resident will lose 24 points).	16
Publishing PubMed ID each additional time per year.	16 bonus
Case log completion in all categories. (Each PGY1–4 resident must meet a 20% multiple of the minimums available to residents at year end or lose 20 pts. PGY1 = 20%; PGY2 = 40%; PGY3 = 60%; PGY4 = 80%. If residents do not meet minimums by December 31, PGY5 residents will lose 40 points. Residents must log at least 200 cases per year or lose 20 pts. Residents will gain 1 pt for each 100 additional cases).	4; bonus
Journal of Neurosurgery (2 points per quarter if all quarterly assigned completed. If residents do not obtain 8 pts in category residents will lose 40 pts for the year.)	8
SANS (2 points per quarter if all quarterly assigned completed. If residents do not obtain 8 pts in category resident will lose 40 pts for the year.)	8
Participation in committees (4 points for four committee attendance per year. If residents do not obtain 2 pts in category resident will lose 40 pts for the year).	2; bonus 2
Attendance at social events (4 points for four social event attendance per year. If residents do not obtain 2 pts in category resident will lose 40 pts for the year).	2; bonus 2
CAP incomplete is a failure for that academic year.	−60
Resident annual survey incomplete.	−50
Resident annual report incomplete by June 28.	−50
Duty hours violations any (residents are responsible; PD will prevent or excuse resident when notified ahead of time).	−30
Maintaining certification (residents will lose 40 pts for every instance that ANY certification lapses one day, e.g., ACLS, BLS, TB/flu annual, second year by December 31 - CA license and DEA; fourth year by December 31 fluoroscopy license; fifth year June 30 ATLS, ACOS, AOA, etc.).	−40
Logging duty hours by two-week due date (20 points will be subtracted for every duty period not meeting deadline).	−20
Resident commendation from program director documented on day of occurrence: 20 points per event if resident obtains at least 290 pts in the year.	20
Resident poor evaluation from program director documented on day of occurrence	−20
TOTAL	300

Residents are expected to embody the highest standards of ethics, professionalism, and self-determination while balancing a rigorous workload that demands technical skill, clinical judgment, and resilience. The RUHS point system functions as both a metric and a mirror, quantifying progress while encouraging reflection on areas needing improvement. By making each component of training transparent and measurable, the system ensures that residents understand not only what is expected of them but also how their performance compares to program standards and milestones.

Quarterly evaluations with the program director incorporate cumulative point totals alongside faculty assessments, providing residents with concrete evidence of performance while offering early warning signals when promotion is at risk. Educational literature supports this proactive model: structured, competency-based assessment systems have been shown to improve fairness, provide more actionable feedback, and enhance both resident self-awareness and program accountability [11]. Systems that combine quantitative metrics with qualitative evaluation foster deliberate practice, a method long recognized as essential for mastery in complex surgical fields [4]. Defined grace periods ensure consistency across the cohort. Minimum thresholds for annual point accumulation at 3, 6, 9, and 12 months provide transparency, while consequences for underperformance reinforce the seriousness of meeting academic and professional standards.

Beyond identifying weaknesses, the system highlights exceptional achievement. Residents who surpass baseline expectations can carry over additional points at the discretion of the program director, rewarding excellence while maintaining rigor. Promotional decisions are not based solely on points - faculty evaluation scores, professionalism, and overall rotation performance remain integral, underscoring that neurosurgical training is ultimately about producing safe, independent, and compassionate physicians.

In summary, the RUHS point system provides a structured framework for measuring progress while supporting growth. It embodies principles of competency-based education by linking performance data to promotion decisions, fostering early identification of strengths and weaknesses, and guiding residents toward mastery of the six ACGME core competencies and the osteopathic principles central to the program’s mission.

After establishing clear expectations through the promotion points system (Table 1), the program uses structured, technology-enabled evaluation to ensure that resident development remains continuous, transparent, and mission-driven. In competency-based medical education, programs that align assessments to explicit outcomes and use longitudinal data for coaching and remediation demonstrate stronger validity, clearer progression decisions, and earlier identification of learners who need targeted support [6].

The RUHS Neurosurgery Residency organizes education around multiple, redundant safety nets, each designed to detect strengths and weaknesses early and translate them into actionable plans. These include milestone-based assessments, structured operative autonomy evaluations, scheduled quizzes and exams linked to the reading curriculum, resident improvement meetings, and structured mechanisms for anonymous reporting of concerns. The intent is to reduce reliance on episodic impressions and instead build a learning system in which resident performance trends are visible, discussed, and acted upon before problems become entrenched [5].

Technology is essential to making this feasible on a scale. Centralized, technology-driven educational platforms allow immediate documentation of evaluations, duty hours, case logs, and professionalism events, and they permit the CCC to review resident trajectories over time rather than single-rotation snapshots [7]. Programs that convert such data into individualized coaching plans can strengthen self-regulated learning, improve accountability, and better align day-to-day training with ACGME Milestones and board requirements.

Neurosurgical education also depends on procedural rehearsal outside the operating room. Evidence from surgical education demonstrates that simulation - including cadaveric dissection, 3D-printed models, and virtual reality - can accelerate early technical acquisition, provide objective metrics for progression, and improve learner confidence while protecting patient safety [12]. When these modalities are integrated with structured feedback, they allow faculty to identify specific technical deficits and prescribe targeted deliberate practice that can be reassessed longitudinally.

Finally, the program recognizes that the clinical learning environment - psychological safety, professional culture, and wellness support - is inseparable from performance. Contemporary graduate medical education (GME) evidence links respectful, inclusive learning climates to improved reporting behaviors, stronger communication, and reduced risk of burnout-related performance lapses [9]. By embedding wellness infrastructure and transparent grievance pathways into routine program evaluation, RUHS treats professionalism and resilience as measurable competencies that can be strengthened through early intervention and coaching.

The residency promotion point system

The implementation of the RUHS residency promotion point system has provided a structured, transparent method to monitor resident development, identify potential weaknesses early, and promote individual strengths. Tracking points in real time and formally reporting quarterly has allowed the program to identify residents who fall behind minimal requirements, ensuring that deficiencies are addressed promptly before they jeopardize long-term performance. When residents fall more than 10% below the quarterly benchmarks, despite counseling from the program director, senior residents, and mentors, they are placed on a structured, goal-oriented success plan.

The RUHS point system has demonstrated effectiveness in two critical ways. First, it identifies residents’ strengths, enabling faculty to direct motivated learners toward leadership, scholarship, professionalism, or subspecialty opportunities. Second, it highlights those who are struggling to meet the rigorous demands of neurosurgical training. Residents who continue to fall short of benchmarks after structured intervention are, after careful review by the CCC, deemed unable to meet the requirements for successful completion of the program. This approach aligns with evidence that early identification of struggling learners and the use of structured remediation improves both learner outcomes and program integrity [7]. Competency-based systems that combine quantitative benchmarks with narrative feedback have been shown to increase fairness, transparency, and defensibility of promotion and dismissal decisions. Point totals are reviewed privately between each resident and the program director during quarterly meetings and are not shared across the cohort, preserving a collaborative rather than competitive learning culture.

By linking promotion to accumulated points, the RUHS system moves beyond subjective impressions toward an evidence-based framework that supports resident success while protecting patients and the public.

The resident quiz and question-writing system

The RUHS neurosurgery residency program has implemented a structured quiz and question-writing system that has proven effective in strengthening medical knowledge while simultaneously identifying residents who may struggle to meet the intellectual and organizational demands of the specialty. Each resident is required to read in advance of weekly didactic sessions and submit two multiple-choice questions, with referenced justifications for each answer choice. Quizzes composed of peer-submitted questions are administered one week following the didactic session, and every other month residents complete a larger exam built from questions not selected for the weekly quizzes.

Tracking compliance and performance within this system has produced several important outcomes. Timely submission of questions has correlated with accountability, organization, and professional responsibility, while missed submissions have consistently identified residents at risk of poor academic performance. Poor quiz performance has been directly correlated with poor performance on the annual in-service training examination, a national benchmark for neurosurgical training. Conversely, residents who excel in quizzes and exams perform strongly on in-service testing, demonstrating the predictive validity of this formative system.

The program has also leveraged the system to promote both remediation and mentorship. Strengths are identified early, and residents who excel in specific topics are invited to mentor their peers, reinforcing mastery through teaching and collaboration. Residents who demonstrate weakness are placed on structured reading plans, provided with additional targeted assessments, and monitored more closely to ensure progressive improvement.

These outcomes align with findings in the medical education literature. Peer-generated question writing is recognized as an effective learning strategy, fostering deeper engagement with content, enhancing critical reasoning, and promoting durable retention of knowledge [13]. Regular low-stakes quizzes have been shown to improve long-term knowledge retention, predict performance on high-stakes examinations, and support early identification of struggling learners [14]. Structured formative assessment programs, when paired with faculty-guided remediation, have demonstrated effectiveness in improving exam outcomes and ensuring academic progression in residency training [15]. Embedding question-writing and frequent testing into the curriculum creates a continuous cycle of preparation, performance, feedback, and targeted improvement.

Statistical testing was performed using Pearson’s correlation coefficients (r-value) to determine the relationship between two variables at a time in this study’s sample. Variables with r-values closer to 1 were determined to have a strong positive relationship, while variables with r-values closer to 0 were determined to have a weak or no relationship. p-values <0.05 were considered statistically significant.

## Results

Correlation between resident point accumulation and academic performance

Table 2 displays the points acquired by the residents each quarter, organized as a rank list with the resident who obtained the most points in a year at the top. Analysis of Table 2 (Points by Quarter) and Table 4 (Quiz and Points Performance Corresponding to Training In-Service Exam Performance) demonstrated several statistically significant relationships between residents’ quarterly point totals and measures of knowledge acquisition, engagement, and standardized exam success.

**Table 2:** Points by quarter.

Resident	Quarter 1	Quarter 2	Quarter 3	Quarter 4	Total points
Resident-15	86	71	163	77	397
Resident-05	69	73	131	85	358
Resident-04	46	83	161	57	347
Resident-08	59	72	148	59	338
Resident-10	70	63	93	73	299
Resident-12	66	66	84	75	291
Resident-13	67	70	96	58	291
Resident-03	42	36	157	47	282
Resident-11	86	65	75	54	280
Resident-02	69	64	77	65	275
Resident-14	70	63	73	57	263
Resident-17	64	38	74	64	240
Resident-16	49	59	55	34	197
Resident-01	46	8	76	60	190
Resident-18	−22	69	72	55	174
Resident-07	41	63	−2	59	161
Resident-09	4	−4	80	65	145
Resident-06	−28	−47	69	52	46

Across all residents (n = 18), Quarter 3 points showed the strongest positive correlation with American Board of Neurological Surgery (ABNS) board scores (r = 0.79, p < 0.001) and a substantial association with American Osteopathic Association (AOA) exam scores (r = 0.61, p = 0.007). Performance in Quarter 2 and Quarter 3 also correlated closely with overall annual totals (r = 0.77 and 0.71, respectively; p < 0.001). Quarter 1 points were significantly related to total yearly performance (r = 0.79, p < 0.001), suggesting that early engagement reliably forecasts later success.

When cumulative totals were examined, total points correlated significantly with both average quiz score (r = 0.55, p = 0.019) and ABNS score (r = 0.52, p = 0.025), indicating that the RUHS promotion-points framework is not merely an administrative metric but a valid educational measure that predicts cognitive and professional performance. Moderate correlations were also observed between Quarter 4 points and questions submitted (r = 0.51, p = 0.030), implying that residents who remain academically engaged near the end of the year sustain productivity and scholarship.

Correlation between quiz performance and standardized outcomes

Table 3 displays the quiz scores expressed as percentage (%) correct of the residents each quarter, organized in the same order of residents as Table 2. Evaluation of Table 3 (Quiz Scores by Quarter) relative to Table 4 confirmed the predictive validity of the resident quiz system.

**Table 3:** Quiz scores by quarter.

Resident	Quarter 1	Quarter 2	Quarter 3	Quarter 4
Resident-15	69%	72%	63%	69%
Resident-05	71%	70%	71%	87%
Resident-04	59%	56%	59%	75%
Resident-08	77%	62%	77%	70%
Resident-10	77%	65%	77%	52%
Resident-12	41%	67%	41%	70%
Resident-13	52%	58%	52%	45%
Resident-03	75%	63%	75%	62%
Resident-11	69%	50%	69%	7%
Resident-02	77%	53%	77%	31%
Resident-14	57%	53%	57%	51%
Resident-17	58%	62%	58%	72%
Resident-16	69%	51%	69%	26%
Resident-01	44%	63%	44%	56%
Resident-18	79%	62%	79%	72%
Resident-07	68%	72%	68%	53%
Resident-09	65%	36%	65%	46%
Resident-06	38%	43%	38%	46%

Early-year performance was a powerful predictor of neurosurgical residency success. Quarter 1 quiz scores correlated strongly with average quiz score (r = 0.71, p < 0.001), demonstrating that early mastery of foundational topics aligns with sustained academic success. Quarter 2 and 3 scores retained significant associations with average quiz score (r = 0.68 and 0.70, respectively; p ≤ 0.002), reinforcing the value of continuous formative assessment. Quarter 4 quiz performance correlated with ABNS score (r = 0.62, p = 0.006), confirming that end-of-year assessments mirror readiness for standardized testing. Questions submitted correlated with Quarter 2 quiz scores (r = 0.65, p = 0.0038) and with overall total points (r = 0.52, p = 0.028), underscoring that residents who participate actively in content creation display higher knowledge retention and stronger overall performance.

Table 4 displays the average quiz scores and points accumulated for the year by each resident alongside their rank performance on the in-training board examinations.

**Table 4:** Quiz and points performance corresponding to in-service training exam performance. ABNS: American Board of Neurological Surgery; AOA: American Osteopathic Association.

Resident	Avg quiz score	Questions submitted	ABNS score	AOA score	Total points	Point rank	Questions submitted rank	ABNS rank	AOA rank	Points rank
Resident-15	68%	28	76%	83	397	5	2	1	1	1
Resident-05	75%	30	72%	76	358	1	1	2	4	2
Resident-04	62%	8	62%	81	347	9	16	4	3	3
Resident-08	72%	28	62%	76	338	3	2	4	4	4
Resident-10	68%	22	23%	61	299	6	6	14	17	5
Resident-12	55%	16	43%	72	291	11	11	8	9	6
Resident-13	52%	20	58%	67	291	16	7	6	12	6
Resident-03	69%	12	69%	82	282	4	13	3	2	8
Resident-11	49%	12	10%	62	280	17	13	17	16	9
Resident-02	60%	10	31%	72	275	10	15	13	9	10
Resident-14	55%	20	3%	45	263	12	7	18	18	11
Resident-17	63%	24	34%	63	240	8	5	11	15	12
Resident-16	53%	8	33%	74	197	13	16	12	7	13
Resident-01	52%	14	42%	73	190	15	12	10	8	14
Resident-18	73%	20	43%	68	174	2	7	8	11	15
Resident-07	65%	28	17%	64	161	7	2	16	13	16
Resident-09	53%	8	46%	76	145	14	16	7	4	17
Resident-06	41%	20	23%	64	46	18	7	14	13	18

Table 5 displays comparisons between several Table 2 variables and Table 4 variables, with Pearson’s correlation coefficients and associated p-values.

**Table 5:** Correlation and statistical significance between Table 2 variables (points per quarter) and Table 4 variables (quiz & points/exam performance). *p-values <0.05 were considered statistically significant. ABNS: American Board of Neurological Surgery.

Table 2 variable ↔ Table 4 variable	r-value	p-value	Interpretation
Q3 ↔ ABNS Score	0.79	p < 0.001*	Strong, statistically significant correlation - high Q3 points strongly predict ABNS performance
Q1 ↔ Total Points	0.79	p < 0.001*	Strong, significant correlation - early quarter performance predicts overall success
Q2 ↔ Total Points	0.77	p < 0.001*	Strong correlation - consistent mid-year point performance predicts annual outcomes
Q3 ↔ Total Points	0.71	p < 0.001*	Strong correlation - major predictive value

Table 6 displays comparisons between several Table 3 variables and Table 4 variables.

**Table 6:** Correlation and statistical significance between Table 3 variables (quiz scores by quarter) and Table 4 variables (quiz & points/exam performance). *p-values <0.05 were considered statistically significant.

Table 3 variable ↔ Table 4 variable	r-value (correlation)	p-value	Interpretation
Q1 ↔ Average Quiz Score	0.71	p < 0.001*	Very strong, highly significant correlation - early quiz performance strongly predicts sustained knowledge performance.
Q3 ↔ Average Quiz Score	0.70	p = 0.0014*	Strong and significant - mid-year quiz mastery maintains predictive validity for long-term success.
Q2 ↔ Average Quiz Score	0.68	p = 0.0018*	Strong, significant - steady mid-year performance remains a reliable indicator.
Q4 ↔ Average Quiz Score	0.65	p = 0.0034*	Strong, significant - end-of-year quizzes track consistent learning gains.
Q2 ↔ Questions Submitted	0.65	p = 0.0038*	Moderate-to-strong - engagement in content creation correlates with improved mid-year scores.

Educational significance

Together, these analyses demonstrate that both the promotion points and quiz systems serve as effective predictive instruments within the RUHS Neurosurgery Residency. Quarterly performance metrics, particularly in the second and third quarters, identify residents’ strengths and weaknesses early, enabling timely mentorship and individualized success plans.

Residents who accumulated higher points and quiz scores were also those achieving superior ABNS and AOA exam results, validating the integration of formative and summative assessments within a competency-based medical education framework. These findings align with broader educational literature showing that continuous formative feedback, active learning, and engagement in self-assessment correlate with improved summative outcomes [10,13,2]. RUHS data extend this evidence to neurosurgical training, confirming that structured performance analytics can both predict and enhance resident competency development.

The strongest predictors of board and exam success are Quarter 3 points, which strongly predict ABNS and AOA scores, aligning with the midpoint of the academic year when clinical autonomy and cognitive load are highest. High correlations between early-year (Q1-Q2) scores and end-of-year outcomes support the predictive validity of the RUHS formative system - an approach endorsed in ACGME Milestones 2.0 and competency-based medical education literature [2,6]. The statistically significant Q4-ABNS correlation (r = 0.62) validates the RUHS internal assessment framework as a meaningful measure of board readiness.

## Discussion

Our program is intentionally built on the philosophy that neurosurgical competence is more than technical performance. It is the integrated development of knowledge, skill, and character in a resident who must function under sustained cognitive load, physical strain, and moral pressure. The RUHS Neurosurgery Residency is designed as a holistic, multifaceted, and continuously measured educational system in which the structure of training drives the function of the graduate. When these elements are aligned and measured in real time, strengths can be amplified, weaknesses can be identified early, and both residents and faculty can be redirected toward shared goals, ACGME Milestones, and outcomes that match the program’s mission. A core finding of this manuscript is that measurable structure improves resident function.

One of the central themes in the RUHS program’s evolution has been the use of structured tools - including promotion points, quizzes, milestone dashboards, and simulation data - not only to monitor performance but also to intervene early. Studies in competency-based medical education emphasize that trainees who receive timely, specific feedback coupled with actionable remediation plans show higher rates of progression toward independence compared with those evaluated by retrospective global judgments alone [16]. By linking quarterly and annual thresholds to promotion, the system provides both residents and faculty with objective markers of growth while also allowing for early redirection if goals are not met.

Beyond identifying weaknesses, the system also highlights strengths that can be leveraged. Residents excelling in certain domains - such as research productivity or communication - are often positioned as peer mentors, an approach supported by peer-assisted learning literature that demonstrates measurable benefits for both the teaching resident and their peers [17].

Predictive technology and anticipatory guidance

The incorporation of predictive technology into neurosurgical training is particularly innovative. Adaptive learning platforms have been shown to detect at-risk learners weeks to months before traditional assessments reveal failure. Translating this approach to surgical residency, our system uses trends from in-service examinations, simulation metrics, and case logs to forecast areas where residents may struggle. Such predictive modeling aligns with proposals from competency-based training experts who advocate for longitudinal, data-driven learning analytics to supplement subjective evaluations [18]. Early results from this integration suggest that predictive dashboards can detect declining performance before complications or failures manifest. Literature from surgical education confirms that simulation and predictive metrics can accelerate learning curves and reduce errors, especially in high-stakes fields like neurosurgery [19].

Simulation and procedural mastery

Simulation has become a cornerstone of modern surgical education, and the RUHS experience reinforces its importance. Cadaveric dissections, 3D printing models, and virtual reality (VR) platforms all serve as adjuncts to operative training. Studies consistently show that VR and 3D simulation improve procedural accuracy, shorten operative times, and enhance confidence among trainees [3]. In neurosurgery, where case volumes may be limited and rare pathologies constrain exposure, simulation offers repeated deliberate practice without risk to patients. Published outcomes from our institution - such as the use of 3D skull and spine models to teach external ventricular drain placement and pedicle screw insertion - align with findings that tactile feedback and anatomic realism are critical to skill transfer [20].

Simulation data provide objective, quantifiable measures of resident progress. Metrics such as time to completion, error rates, and motion economy correlate with operative performance and can be trended longitudinally [15]. When integrated with promotion points and milestone evaluations, these data help identify residents progressing more slowly than expected, allowing for remediation before patient safety is compromised.

Faculty engagement and development

No system of resident education can succeed without faculty who are motivated, skilled, and supported in their teaching roles. Faculty development literature demonstrates that programs which offer structured educator training, mentorship opportunities, and recognition for teaching contributions achieve better outcomes in both resident satisfaction and faculty engagement [21]. The RUHS program’s five-year faculty development plan, which emphasizes reflective teaching, feedback training, and osteopathic integration, exemplifies these principles.

Professionalism, wellness, and holistic development

Professionalism remains a core competency, and its integration with wellness monitoring represents an innovative step forward. Evidence shows that burnout and fatigue are associated with lapses in professionalism, errors, and attrition in residency [9]. RUHS’ use of duty hour tracking, wellness surveys, and point systems directly links professional accountability to well-being. Residents unable to meet expectations despite counseling are placed on goal-oriented success plans, a process supported by remediation frameworks that emphasize transparency, documentation, and mentorship [22]. Programs that incorporate resilience training, peer support networks, and structured wellness curricula report lower burnout rates and greater resident satisfaction [22].

Diversity, competency, equity, and recruitment

The structured interview and holistic recruitment process ensures that the residents RUHS matches embody not only intellectual rigor but also resilience, empathy, communication skills, and cultural humility. Literature consistently demonstrates that structured interviews improve reliability and fairness compared to unstructured formats [23]. Furthermore, team diversity has been linked to improved surgical outcomes and patient satisfaction. Evidence shows that inclusive training environments not only improve team function but also better prepare residents to care for diverse patient populations [24].

Global neurosurgical shortages and workforce implications

Globally, the shortage of neurosurgeons remains profound, with estimates suggesting that low- and middle-income countries face deficits of thousands of specialists [25]. Simulation, predictive analytics, and structured evaluation systems could be adapted to resource-limited settings, allowing for distributed training models that scale expertise where case exposure is limited. The literature highlights that task-sharing, modular training, and use of digital platforms have already begun to expand access to neurosurgical care in underserved areas [26].

Long-term program outcomes and alumni tracking

The ultimate measure of a residency program is the performance of its graduates. Evidence from other surgical specialties shows that programs with structured faculty development, longitudinal assessment, and simulation-rich curricula produce graduates with higher board pass rates, greater scholarly productivity, and lower rates of disciplinary action [27]. Alumni outcome tracking, including board certification rates, practice patterns, and peer assessments of independent competence, represents a critical next step and is currently being developed as a formal quality improvement initiative at RUHS. This aligns with recommendations that GME programs measure downstream effects on patient care and workforce needs [28].

Integrating systems toward a unified mission

Taken together, these elements - structured assessments, predictive analytics, simulation, faculty development, wellness initiatives, inclusive recruitment, and alumni tracking - form a coherent system designed to achieve the program’s mission of producing safe, independent, and compassionate neurosurgeons. The RUHS promotion points model appears to be a novel, competency-aligned approach in neurosurgery that operationalizes quarterly, threshold-based early-warning signals linked to structured remediation and CCC decision-making, enabling earlier identification of both strengths and emerging risks to successful progression.

While competency-based medical education frameworks are employed broadly across ACGME-accredited programs regardless of osteopathic recognition, the RUHS model incorporates osteopathic principles, including whole-person care, mind-body-spirit integration, and manual medicine evaluation, as explicit point-bearing activities. This distinguishes it from purely allopathic frameworks and reflects the program's dual accreditation mission. Future comparative studies examining outcomes between osteopathic-recognized and non-osteopathic neurosurgery programs would help clarify whether these structural differences produce measurable differences in graduate competency profiles.

Study limitations

There are several limitations to this study. This was a retrospective study performed at a single institution. Other neurosurgical residency programs may have different requirements and metrics for their own residents. Potential downsides of a point-based system include the risk of residents optimizing for point accumulation rather than genuine skill development, and the administrative burden of longitudinal tracking. Programs adopting this model should ensure that faculty maintain qualitative mentorship relationships that contextualize quantitative metrics. Additionally, the quantitative analyses presented here primarily reflect knowledge acquisition and engagement metrics. While simulation and operative autonomy data are collected, the heterogeneity of these assessments across training levels precluded their inclusion in the current correlational analysis. Future studies should incorporate multivariate models integrating technical skill metrics and milestone-based autonomy assessments alongside knowledge performance indicators.

## Conclusions

The RUHS neurosurgery resident promotion points system represents a novel application in neurosurgery training by integrating clinical performance requirements, administrative reliability, professional behaviors, and academic contribution into a single structured currency that functions as both an accountability mechanism and an early-warning indicator. By coupling quarterly point thresholds with predefined escalation pathways and success planning, the system operationalizes competency-based education into a measurable and actionable framework that promotes early intervention, reinforces professional standards, and improves downstream educational outcomes. A core finding of this manuscript is that measurable structure improves resident function.

The point system drives organizational performance and professionalism; the quiz system drives deliberate cognitive preparation; simulation advances technical mastery; wellness systems protect emotional resilience; faculty development enhances teaching fidelity; and mission-aligned selection shapes the incoming cohort. These domains are connected - a resident who is overwhelmed organizationally will underperform cognitively; a resident who is fatigued will struggle with communication; a resident who lacks psychological safety will withhold concerns and miss learning opportunities. By designing and measuring holistic structure intentionally, RUHS demonstrates that neurosurgery education can not only meet but also exceed accreditation standards, producing graduates who are technically excellent, intellectually rigorous, emotionally resilient, and professionally grounded.

Future work should employ multivariate analyses integrating knowledge metrics, simulation-derived technical skill data, and milestone-based assessments of non-technical skills, including communication, professionalism, and systems-based practice, to more fully characterize the determinants of neurosurgical resident success within structured competency frameworks.
